# Supplementary material for: Drug repurposing for aging research using model organisms
Source: Aging Cell. 2017 Jun 16;16(5):1006–15. doi: 10.1111/acel.12626 (PMC5595691; doi:10.1111/acel.12626)
Supplement: Supplementary file 7 — Data S1 Zip‐Archive of all report cards. [file ACEL-16-1006-s007.zip › RC_ORN.pdf]

ORN

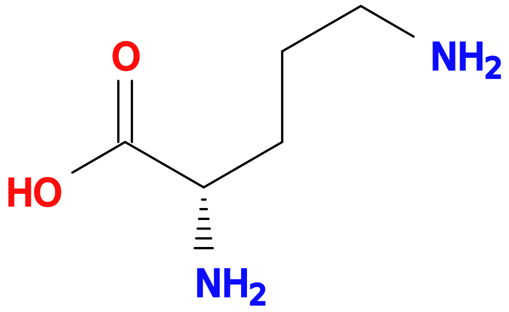

Database identifiers

ChEMBLCompound	CHEMBL446143
DrugBank	DB00129
CHEBI	15729
eMolecules	884260

Ranking

	Rank	Score
Drosophila	485/697	0.292
C. elegans	NA	NA

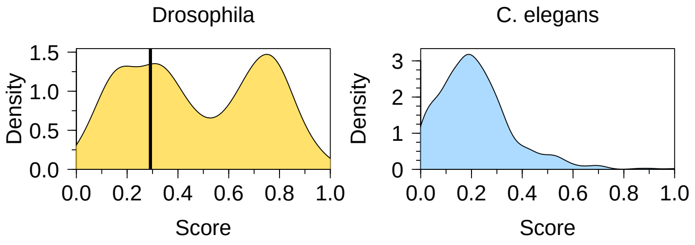

	Ageing implication	Domain conservation	Binding site conservation	Binding affinity	Bioavailability	Lipinski	Promiscuity	Purchasability	Drug approval	**Total**
Drosophila	0.624	0.802	0.922	0.284	(0.9)	0.0	-0.001	0.1	0.075	0.292
C. elegans	NA	NA	NA	NA	NA	NA	NA	NA	NA	NA

Names

- **L-ornithine**
- (S)-2,5-Diaminopentanoate
- (S)-2,5-Diaminopentanoic acid
- (S)-2,5-diaminovaleric acid
- (S)-alpha,delta-diaminovaleric acid

Roles

algal metabolite, hepatoprotective agent

Status

Approved drug (according to ChEMBL)	No
Classification (according to DrugBank)	approved, nutraceutical
Administration Route	Oral
Number of Rule of 5 violations	0
Binding affinity to original target in log units (RF-Score prediction)	4.07
Burns *C. elegans* bioavailability prediction	-3.27

Medical Information

Indicatation: Used for nutritional supplementation, also for treating dietary shortage or imbalance. It has been claimed that ornithine improves athletic performance, has anabolic effects, has wound-healing effects, and is immuno-enhancing.
Mechanism of action: L-Ornithine is metabolised to L-arginine. L-arginine stimulates the pituitary release of growth hormone. Burns or other injuries affect the state of L-arginine in tissues throughout the body. As De novo synthesis of L-arginine during these conditions is usually not sufficient for normal immune function, nor for normal protein synthesis, L-ornithine may have immunomodulatory and wound-healing activities under these conditions (by virtue of its metabolism to L-arginine).
Toxicity: Oral, rat LD50 = 10000 mg/kg
Metabolism: Ornithine undergoes extensive metabolism in the liver to L-arginine, polyamines, and proline, and several other metabolites.

(Information from DrugBank)

Compound Target Characteristics

Arginase-1

Best gene implication in ageing for this target family came from gene P07824 via mapping the annotation from RGD 2150 annotated in RGD 2014-03-11. Annotation GO 7568 (aging) was Inferred from Expression Pattern

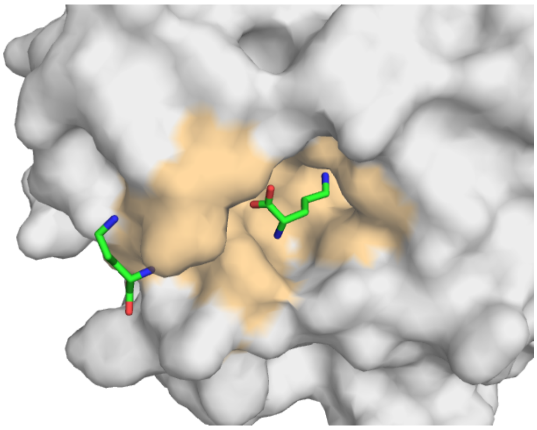

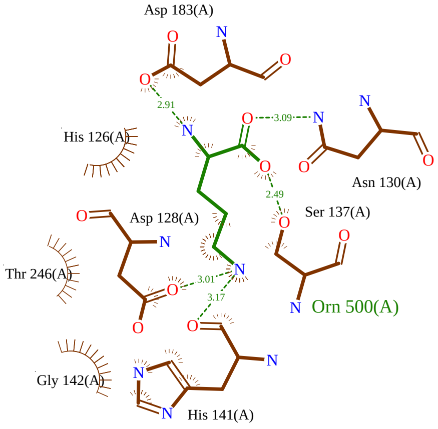

protein	amino acids contacts (binding site)
PDB:3gmz:chainA:P05089	H D N T T T S H G D E T
PDB:3thj:chainA:P05089	H D N T T T S H G D E T
sp:P05089:ARGI1_HUMAN	H D N T T T S H G D E T
sp:P07824:ARGI1_RAT	H D N T T S S H G D E T
sp:Q61176:ARGI1_MOUSE	H D N T T S S H G D E T
tr:Q9NHA5:Q9NHA5_DROME	H D N T S Q S H G D E T
tr:O76895:O76895_DROME	H D N T S Q S H G D E T
sp:P00812:ARGI_YEAST	H D N S T P S H G D E T

	whole protein		domain-based		contact-based	
protein	ident	simil	ident	simil	ident	simil
PDB:3gmz:chainA:P05089	1.0	1.0	1.0	1.0	1.0	1.0
PDB:3thj:chainA:P05089	1.0	1.0	1.0	1.0	1.0	1.0
sp:P05089:ARGI1_HUMAN	1.0	1.0	1.0	1.0	1.0	1.0
sp:P07824:ARGI1_RAT	0.87	0.96	0.87	0.96	0.92	0.95
sp:Q61176:ARGI1_MOUSE	0.87	0.96	0.87	0.96	0.92	0.95
tr:Q9NHA5:Q9NHA5_DROME	0.35	0.71	0.36	0.74	0.83	0.92
tr:O76895:O76895_DROME	0.34	0.7	0.36	0.74	0.83	0.92
sp:P00812:ARGI_YEAST	0.39	0.71	0.4	0.72	0.83	0.93

arg (FBgn0023535) associated phenotypes
partially lethal - majority die, some die during pupal stage

(Information from FlyBase)

arg (UniProt:Q9NHA5) annotation
Cofactor: Mn(2+) Evidence=(RuleBase:RU361159); Note=Binds 2 manganese ions per subunit. (RuleBase:RU361159);
Pathway: Nitrogen metabolism; urea cycle; L-ornithine and urea from L-arginine: step 1/1. (RuleBase:RU361159).

(Information from UniProt)

arg (UniProt:O76895) annotation
Cofactor: Mn(2+) Evidence=(RuleBase:RU361159); Note=Binds 2 manganese ions per subunit. (RuleBase:RU361159);
Pathway: Nitrogen metabolism; urea cycle; L-ornithine and urea from L-arginine: step 1/1. (RuleBase:RU361159).

(Information from UniProt)

Arginase-1

Best gene implication in ageing for this target family came from gene P07824 via mapping the annotation from RGD 2150 annotated in RGD 2014-03-11. Annotation GO 7568 (aging) was Inferred from Expression Pattern

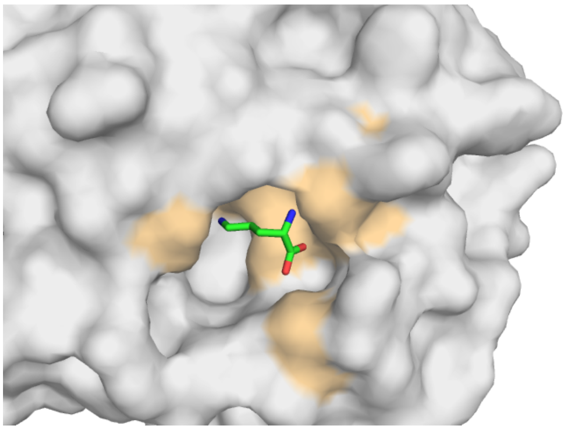

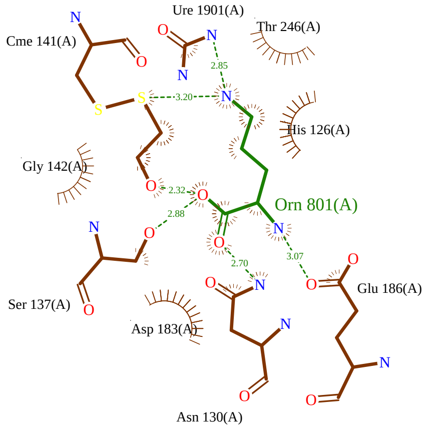

protein	amino acids contacts (binding site)
PDB:1hqg:chainA:P07824	H D N S X G D E T
sp:P05089:ARGI1_HUMAN	H D N S H G D E T
sp:P07824:ARGI1_RAT	H D N S H G D E T
sp:Q61176:ARGI1_MOUSE	H D N S H G D E T
tr:Q9NHA5:Q9NHA5_DROME	H D N S H G D E T
tr:O76895:O76895_DROME	H D N S H G D E T
sp:P00812:ARGI_YEAST	H D N S H G D E T

	whole protein		domain-based		contact-based	
protein	ident	simil	ident	simil	ident	simil
PDB:1hqg:chainA:P07824	1.0	1.0	1.0	1.0	1.0	0.89
sp:P05089:ARGI1_HUMAN	0.87	0.96	0.87	0.96	0.89	0.91
sp:P07824:ARGI1_RAT	1.0	1.0	1.0	1.0	0.89	0.91
sp:Q61176:ARGI1_MOUSE	0.94	0.98	0.93	0.98	0.89	0.91
tr:Q9NHA5:Q9NHA5_DROME	0.33	0.71	0.35	0.74	0.89	0.91
tr:O76895:O76895_DROME	0.33	0.7	0.35	0.74	0.89	0.91
sp:P00812:ARGI_YEAST	0.37	0.71	0.38	0.72	0.89	0.91

arg (FBgn0023535) associated phenotypes

partially lethal - majority die, some die during pupal stage

(Information from FlyBase)

arg (UniProt:Q9NHA5) annotation

Cofactor: Mn(2+) Evidence=(RuleBase:RU361159); Note=Binds 2 manganese ions per subunit. (RuleBase:RU361159);

Pathway: Nitrogen metabolism; urea cycle; L-ornithine and urea from L-arginine: step 1/1. (RuleBase:RU361159).

(Information from UniProt)

arg (UniProt:O76895) annotation

Cofactor: Mn(2+) Evidence=(RuleBase:RU361159); Note=Binds 2 manganese ions per subunit. (RuleBase:RU361159);

Pathway: Nitrogen metabolism; urea cycle; L-ornithine and urea from L-arginine: step 1/1. (RuleBase:RU361159).

(Information from UniProt)
